# Case report: Optical genome mapping revealed double rearrangements in a male undergoing preimplantation genetic testing

**DOI:** 10.3389/fgene.2023.1132404

**Published:** 2023-03-31

**Authors:** Jun Ren, Yuezhi Keqie, Yutong Li, Lingping Li, Min Luo, Meng Gao, Cuiting Peng, Han Chen, Ting Hu, Xinlian Chen, Shanling Liu

**Affiliations:** ^1^ Center of Prenatal Diagnosis, Department of Medical Genetics, West China Second University Hospital, Sichuan University, Chengdu, China; ^2^ Department of Obstetrics and Gynecology, West China Second University Hospital, Sichuan University, Chengdu, China; ^3^ Key Laboratory of Birth Defects and Related Diseases of Women and Children (Sichuan University), Ministry of Education, Chengdu, China

**Keywords:** optical genome mapping, PGT-SR, complex chromosome rearrangements, cryptic reciprocal translocation, FISH

## Abstract

Chromosome rearrangement is one of the main causes of abortion. In individuals with double chromosomal rearrangements, the abortion rate and the risk of producing abnormal chromosomal embryos are increased. In our study, preimplantation genetic testing for structural rearrangement (PGT-SR) was performed for a couple because of recurrent abortion and the karyotype of the male was 45, XY der (14; 15)(q10; q10). The PGT-SR result of the embryo in this *in vitro* fertilization (IVF) cycle showed microduplication and microdeletion at the terminals of chromosomes 3 and 11, respectively. Therefore, we speculated whether the couple might have a cryptic reciprocal translocation which was not detected by karyotyping. Then, optical genome mapping (OGM) was performed for this couple, and cryptic balanced chromosomal rearrangements were detected in the male. The OGM data were consistent with our hypothesis according to previous PGT results. Subsequently, this result was verified by fluorescence *in situ* hybridization (FISH) in metaphase. In conclusion, the male’s karyotype was 45, XY, t(3; 11)(q28; p15.4), der(14; 15)(q10; q10). Compared with traditional karyotyping, chromosomal microarray, CNV-seq and FISH, OGM has significant advantages in detecting cryptic and balanced chromosomal rearrangements.

## Introduction

Complex chromosomal rearrangements (CCRs) are rare structural rearrangements. CCRs generally refer to more than two chromosomes with three or more cytogenetic breakpoints (Ou et al., 2020). The incidence of CCRs in neonates is approximately 0.5% (Durmaz et al., 2016; Hu et al., 2018), and in 3.5% of couples with a history of recurrent abortion, at least one partner was a carrier of structural chromosomal rearrangements (Daya and Stephenson, 1996; Ou et al., 2020). The simplest CCR is the double two-way exchange, in which there are two independent simple reciprocal translocations (Kim et al., 2011). It could also be called double rearrangement (Madan, 2013; Pierron et al., 2019). CCR carriers have a higher risk of unbalanced chromosomes developing in their gametes (Escudero et al., 2008). Therefore, the risk of spontaneous abortion or abnormal foetuses is increased (Vanneste et al., 2011).

Generally, cryptic balanced translocation is difficult to detect by karyotyping, which can only detect fragments larger than about 4 Mb. Moreover, common molecular genetic techniques, such as chromosomal microarray and CNV-seq, cannot detect balanced translocation, although their resolution is greatly improved. In general, female carriers are suspected of having cryptic translocation when genetic causes of recurrent spontaneous abortion are sought or when chromosome abnormalities are found in prenatal diagnosis. Male carriers may also be diagnosed by fertility decline, azoospermia or oligoasthenoteratospermia (Nguyen et al., 2015).

In this study, we report a case of a male who had a Robertsonian translocation accompanied by a cryptic reciprocal translocation. The karyotype of this male was determined to be 45,XY,der(14; 15) (q10; q10) through G-banding. To solve the problem of Robertsonian translocation inheritance and recurrent abortion, the couple underwent IVF and PGT-SR to select a balanced embryo for transfer. In this cycle, one embryo was obtained for PGT-SR, and the PGT-SR revealed the possibility of cryptic translocations of chromosomes 3 and 11. Therefore, OGM was performed for this couple, and FISH analysis was used to verify the OGM results. In conclusion, the male’s karyotype showed double chromosomal rearrangements. Our study provides useful information for the subsequent reproductive and genetic counselling of this couple.

## Patients and methods

### Patients

This couple visited the Department of Medical Genetics (West China Second University Hospital, Sichuan University) because the karyotype of the husband showed a Robertsonian translocation. The couple had naturally conceived a phenotypically healthy offspring (karyotyping and other genetic testing was not performed). The female had not been able to conceive naturally for more than a year without contraception (G4P1+3). The sperm concentration of the male was 79.3*10^6^/mL, 15% were grade A sperm, 15% were grade B sperm, and the proportion of sperm with normal morphology was 1.9%. Most of the sperm were amorphous head sperm. The present study was approved by the Ethics Committee of West China Second University Hospital of Sichuan University. All patients provided written informed consent.

### Preimplantation genetic testing for structural rearrangement

Intracytoplasmic sperm injection (ICSI), trophectoderm biopsy, and embryo transfer were carried out at the Center of Reproductive Medicine (West China Second University Hospital, Sichuan University). Sample collection, whole genome amplification (WGA), library preparation, next-generation sequencing (NGS) and data analysis were conducted at the Department of Medical Genetics (West China Second University Hospital, Sichuan University). Multiple annealing and looping-based amplification cycles (MALBAC) (Yikon Genomics, Soochow, China) were applied for WGA. The WGA product was used for copy number variation (CNV) library preparation *via* an NGS library preparation kit (Yikon Genomics). Library sequencing was performed using the Nextseq CN500 instrument (Illumina, San Diego, CA, United States). For CNV analysis, sequencing files were disposed in the ChromGo (Yikon Genomics) software.

### Optical genome mapping

Ultrahigh molecular weight DNA was isolated from the patients *via* the SP Blood and Cell Culture DNA Isolation Kit (Bionano Genomics, San Diego, CA, United States). Subsequently, the DLS DNA Labeling Kit (Bionano Genomics) was used to fluorescently label long molecules at specific sequence motifs throughout the genome with the enzyme DLE-1 (Bionano Genomics). Labelled DNA was loaded on a Saphyr chip and imaged on the Saphyr instrument for collection of 1,300 Gb of molecules >150 kb. For all samples, a minimum of 320 Gb of data was acquired. The observed unique patterns on single long DNA molecules were used for *de novo* genome assembly and variant annotation with Bionano Solve software v.3.5 (Bionano Genomics). Reporting and direct visualization of structural variants were performed with Bionano Access software (version 1.7.1) (Bionano Genomics). SVs were identified relative to the human reference genome GRCh38/hg38 using the default filter setting (Insertion: 0, Deletion: 0, Inversion: 0.7, Duplication: −1, Intrafusion: 0.05, Inter-translocation: 0.05) (Mantere et al., 2021), and only rare structural variations larger than 5 kb and absent from the Bionano control sample database were considered. DLE markers closest to the SV region defined the boundary of the SV. SV default filters were set at 1% in the control database and compared to an OGM dataset of 204 human population control samples from apparently healthy individuals to filter out common SVs and potential artefacts (both technical and reference genome-related). CNV calls were output and annotated with confidence scores (set at 0.99). Feature CNV overlap precision was over 500 kb (Dremsek et al., 2021; Mantere et al., 2021). Of note, the software refers to duplications that are smaller than 30 kb “insertions” because the label density may not be informative enough to exactly determine the origin of the inserted material. Inversions involving segments of 5 Mb or larger are called “intrachromosomal translocations.”

### Fluorescence *in situ* hybridization, FISH

FISH analysis was performed for the male to further verify the OGM results. The procedure was performed in metaphase in line with the manufacturer’s instructions. Vysis CEP 11 (D11Z1) Spectrum Aqua (Abbott, Chicago, IL, United States), Tel Vysion 3p Spectrum Green (Abbott) and TelVysion 3q Spectrum Orange (Abbott) probes were used for the metaphase FISH analysis. All operations followed the manufacturer’s protocols.

## Results

**FIGURE 1:**
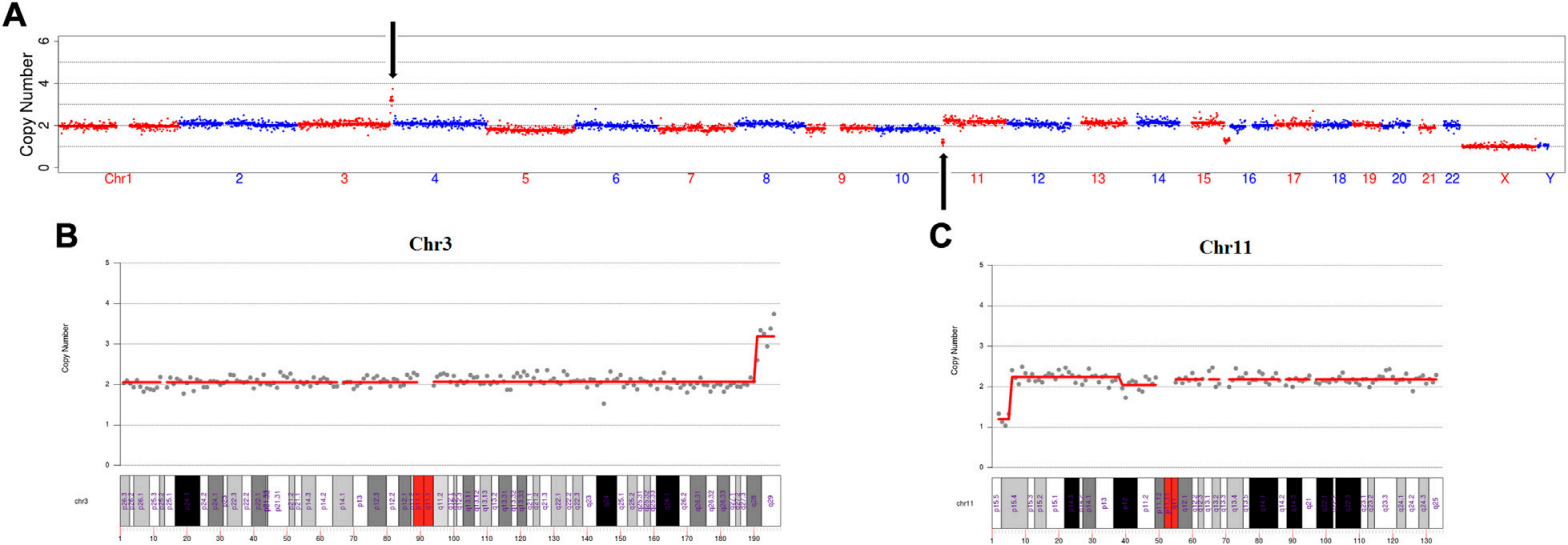
The PGT-SR result revealed a cryptic reciprocal translocation between chromosomes 3 and 11. **(A)** Scatter diagram of the copy number variation of the embryo. This diagram shows that chromosomes 3, 11, and 15 have copy number variations. **(B)** Scatter diagram of the copy number variation of chromosome 3. This diagram clearly shows that microduplication occurred at q28 to q29, which is at the terminus of the long arm of chromosome 3. **(C)** Scatter diagram of the copy number variation of chromosome 11. This diagram explicitly shows that the microdeletion occurred at p15.5 to p15.4, which is at the terminus of the short arm of chromosome 11.

In this IVF cycle, one embryo was biopsied for PGT-SR. The PGT-SR result showed that microduplication of chromosome 3(q28→q29, 6 Mb), microdeletion of chromosome 11(p15.5→p15.4, 4 Mb) and mosaic microdeletion of chromosome 15(q26.1→q26.3, 11 Mb, mosaic ratio about 70%) (Figure 1A). We found that the microduplication of chromosome 3 and the microdeletion of chromosome 11 were located at the termini of the long arm and short arm, respectively (Figure 1B, C). Therefore, we wondered if this couple had cryptic balanced chromosomal rearrangements. To test our hypothesis, OGM was performed for this couple.

**FIGURE 2:**
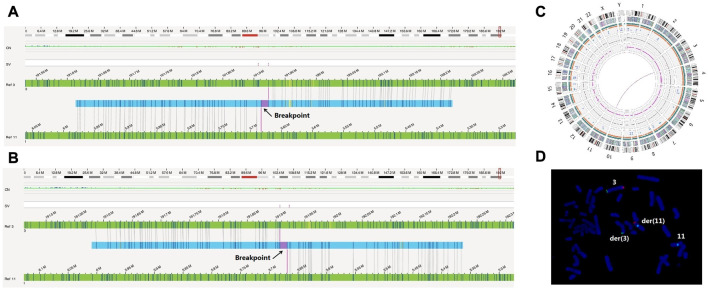
OGM and FISH verified the cryptic reciprocal translocation between chromosomes 3 and 11. **(A)** and **(B)** These two images show the derived chromosomes 3 and 11 compared to the reference chromosomes, respectively. **(C)** This graph directly shows that chromosomes 3 and 11 exhibited a balanced translocation. **(D)** The FISH image clearly shows the occurrence of derived chromosomes and translocation. The green signals (Tel 3p) were located on der (3) and chromosome 3. The orange signals (Tel 3q) were located on der(11) and chromosome 3. The aqua signals (CEP11) were located on der(11) and chromosome 11.

The OGM results revealed that the male had translocation t(3; 11)(q28; p15.4) (Figures 2A–C); no abnormalities were found in the female (data not shown). Due to the limitations of OGM, a Robertsonian translocation was not detected in the male (Figures 2A–C). The karyotype of the male was 45, XY der (14; 15)(q10; q10) by G-banding (data not shown). Obviously, the detection results of OGM were basically consistent with those of PGT-SR for chromosomes 3 and 11.

To verify this finding, fluorescence *in situ* hybridization was performed for the male. A derivative chromosome 11 (CEP11, Aqua) with a 3q (Tel 3q Orange) translocation and a derivative chromosome 3 (Tel 3p Green) without 3q (tel 3q Orange) were translocated to chromosome 11 (Figure 2D). Due to the probe number limitations, the FISH results did not show an 11p translocation fragment, which should have been translocated to derivative chromosome 3. No chromosome rearrangement was found on the other chromosomes 3 (Tel 3p Green, Tel 3q Orange) and 11 (CEP11, Aqua) (Figure 2D).

Combining the karyotyping, OGM and FISH results, the veritable karyotype of this male was 45, XY, t(3; 11)(q28; p15.4), der(14; 15) (q10; q10). His karyotype was a rare and complex Robertsonian translocation accompanied by reciprocal translocation.

## Discussion

In the general population, balanced chromosomal rearrangements which include reciprocal translocation and Robertsonian translocation have a prevalence of about 1/500 (Pierron et al., 2019). The prevalence in male with azoospermic or severe oligozoospermia, and couples with a history of IVF failure and recurrent miscarriage reaching 1.4% and 2.2%, respectively (Clementini et al., 2005; Liu et al., 2013; Zhang et al., 2018). Balanced chromosomal rearrangements carriers are mostly normal phenotypes because there is no numerical loss or gain of genetic material (Zhang et al., 2018). However, carriers usually face sterility problems due to the production of unbalanced gametes, which relate to infertility, recurrent spontaneous abortion or pregnancies with congenital abnormalities (Fiorentino et al., 2011; Zhang et al., 2018). The unbalanced gametes are mainly caused by the segregation patterns of the quadrivalent (Zhang et al., 2018).

During the pachytene stage of meiosis I, the two pairs of homologous centromeres form a quadrivalent with matching of homologous regions (Scriven et al., 1998). At the end of meiosis I, the centromere separate and the chromosomes are moved to the poles by the traction of the spindle fibers. Theoretically, there are five separation models. The 2:2 disjunction of homologous centromeres to opposite poles involve alternate and adjacent-1 segregation modes. On the contrary, when homologous centromeres move to the same pole, the possible separation modes may be adjacent-2, 3:1 or 4:0 disjunctions (Scriven et al., 1998; Zhang et al., 2018). Therefore, theoretically 32 gametes can be formed. However, only the gametes formed by alternate segregation are normal or balanced. Based on theoretical segregation models, translocation carriers have a low probability of forming normal or balanced gametes.

The incidence of double rearrangement in the population is low, but the abortion rate and the risk of producing abnormal chromosomal embryos are relatively high in carrier individuals. Therefore, PGT-SR could lead to better pregnancy outcomes with reduced spontaneous miscarriage rates (Lim et al., 2008; Brunet et al., 2018), and prenatal diagnosis should be carried out in cases of natural pregnancy (Giardino et al., 2009; Ou et al., 2020).

In our study, through karyotyping, PGT-SR, OGM and FISH technology, we ultimately determined that the male had a Robertsonian translocation accompanied by a reciprocal translocation, and his karyotype was 45,XY, t(3; 11)(q28; p15.4), der (14; 15) (q10; q10). This conclusion provides effective information for procreation guidance and genetic counselling for this couple.

Karyotyping is still one of the first-line methods for detecting balanced translocations, but it also has some disadvantages, such as low resolution and the inability to detect reciprocal translocations of smaller fragments (usually fragments larger than approximately 4 Mb can be found). However, molecular genetic techniques, such as chromosomal microarray and CNV-Seq, can only detect unbalanced translocations, although their resolution is greatly improved (Giardino et al., 2006). FISH technology has difficulty detecting balanced translocations of the whole chromosome set due to the restriction of the number of probes and the necessity of knowing loci *a priori* (Zhang et al., 2022). In recent years, OGM has become a very promising method to detect large-scale structural variations in the human genome (Dremsek et al., 2021). The emergence of OGM technology has greatly improved the detection rate of small and cryptic fragment translocations and it has great application prospects in patients with unexplained recurrent spontaneous abortion.

Nonetheless, OGM technology still has some limitations at present. For instance, Robertsonian translocations and other whole-arm translocations that involve the centromere cannot be detected until now (Dremsek et al., 2021). All in all, OGM technique still has outstanding advantages in the diagnosis of chromosome structural variation such as CCR, and can be used as a powerful supplement for karyotyping. Therefore, OGM has application potential in the reproductive genetic fields such as the diagnosis of agnogenic recurrent spontaneous abortion.

## Data Availability

The datasets for this article are not publicly available due to concerns regarding participant/patient anonymity. Requests to access the datasets should be directed to the corresponding authors.
